# MRI Utilization Rates for Veterans at Risk of Prostate Cancer

**DOI:** 10.1001/jamanetworkopen.2025.43567

**Published:** 2025-11-18

**Authors:** Mitchell M. Huang, Joseph D. Nicolas, Ridwan Alam, Nicole Handa, Yutai Li, Clayton Neill, Ashley E. Ross, David J. Bentrem, Frances M. Weaver, John T. Leppert, Ted A. Skolarus, Julie A. Lynch, Adam B. Murphy, Hiten D. Patel

**Affiliations:** 1 Department of Urology, Feinberg School of Medicine, Northwestern University, Chicago, Illinois; 2 Surgery Service, Jesse Brown Veterans Affairs Medical Center, Chicago, Illinois; 3 Department of Surgery, Feinberg School of Medicine, Northwestern University, Chicago, Illinois; 4 Parkinson School of Health Sciences and Public Health, Loyola University, Maywood, Illinois; 5 Center of Innovation for Complex Chronic Healthcare, Edward Hines Jr Veterans Affairs Hospital, Hines, Illinois; 6 Division of Urology, Veterans Affairs Palo Alto Health Care System, Palo Alto, California; 7 Department of Urology, Stanford University School of Medicine, Stanford, California; 8 Department of Surgery, University of Chicago Medicine, Chicago, Illinois; 9 Salt Lake City Veterans Affairs, Veterans Affairs Informatics and Computing Infrastructure, Salt Lake City, Utah; 10 Division of Epidemiology, University of Utah School of Medicine, Salt Lake City

## Abstract

This cross-sectional study evaluates trends in the utilization of and disparities in prostate magnetic resonance imaging (MRI) across the Veterans Health Administration.

## Introduction

Magnetic resonance imaging (MRI) reduces overdiagnosis of low-risk prostate cancer (PCa) while maintaining or increasing detection of high-risk PCa.^1,2^ Surveillance, Epidemiology, and End Results (SEER)–Medicare studies reported a less than 10% utilization rate for MRI in 2015, which increased to 30% through 2019 despite persistent racial, ethnic, and geographic disparities.^3,4^ We hypothesized that as an equal access and integrated health system, the Veterans Health Administration (VHA) might have more equitable use of prebiopsy prostate MRI.

## Methods

The Jesse Brown Veterans Affairs Medical Center institutional review board approved use of national claims and laboratory data from the VHA’s Corporate Data Warehouse (CDW). With *International Statistical Classification of Diseases and Related Health Problems, Tenth Revision* codes and prostate-specific antigen (PSA) laboratory values, we identified biopsy-naive patients with elevated PSA levels who underwent biopsy between January 1, 2015, and December 31, 2023. We used *Current Procedural Terminology* codes to identify veterans receiving prebiopsy MRI. Primary residences were categorized as rural or nonrural (Federal Office of Rural Health Policy’s census tract classification). Race and ethnicity in the CDW are patient reported and routinely recorded to assess health equity and identify potential disparities. We followed the STROBE reporting guideline.

Our primary aim was to identify trends in prostate MRI utilization. Our secondary aim was to identify disparities by race and ethnicity and by rural vs nonrural residence. By-year comparisons used the Fisher exact test. For prebiopsy MRI, multivariable mixed-effects logistic regression was performed with *P* values calculated using asymptotic Wald tests. VHA site was included as a random effect; all other variables were fixed effects. Statistical analysis was performed in R, version 4.4.2 with *P* < .05 considered statistically significant using 2-sided tests. *P* values are exploratory and should not be interpreted as independent confirmatory tests.

## Results

Of 120 105 veterans (median age, 72 years [IQR, 66-76 years]; 1687 Asian or Pacific Islander [1%]; 36 521 Black [30%], 6660 Hispanic or Latino [6%], 70 896 White [59%], and 4321 other race or ethnicity [4%]) who underwent biopsy for elevated PSA level, 33 062 (28%) had a rural primary residence. The prebiopsy MRI utilization rate increased from 2% (121 of 7199) in 2015 to 41% (5885 of 14 244) in 2023, with lower rates for Black and rural veterans (Figure). MRI utilization varied by site; 42% (55 of 132) sites had a prebiopsy MRI utilization rate less than 5%. VHA sites treated a median of 2235 patients (range, 219-9066 patients per site) with elevated PSA levels.

**Figure.**
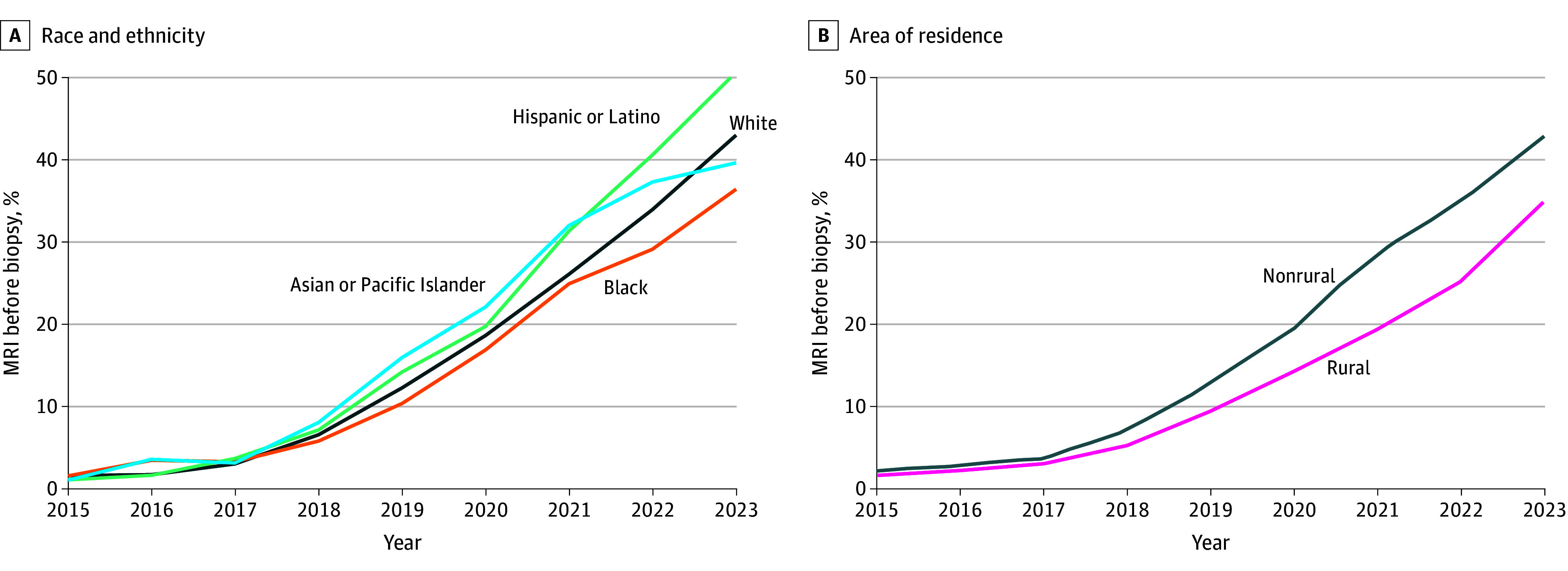
Trends in Rate of Magnetic Resonance Imaging (MRI) Utilization Prior to Prostate Biopsy by Year, 2015-2023, Stratified by Self-Identified Race and Ethnicity (A) and Area of Residence (B) (N = 120 105)

In 2023, 37% of Black veterans (1758 of 4809) vs 44% of veterans who did not identify as Black (4127 of 9435) received prebiopsy MRI (−7% absolute and −16% relative; *P* < .001), despite Black patients being younger (median age, 68 [IQR, 63-73] years compared with 72 [IQR, 66-76] years; *P* < .001) and having higher PSA levels (>10 ng/mL, 14% [654 of 4809] compared with 11% [1019 of 9435]; *P* < .001). The MRI utilization rate by 2023 was 35% (1362 of 3884) for rural patients vs 43% (4723 of 11 011) for nonrural patients (−8% absolute and −19% relative; *P* < .001). In adjusted models, Black and rural veterans had lower odds of receiving prebiopsy MRI across the time period (Table) and in 2023 (Black compared with White veterans: odds ratio [OR], 0.83 [95% CI, 0.75-0.91]; *P* < .001; rural vs nonrural veterans: OR, 0.73 [95% CI, 0.67-0.81]; *P* < .001).

**Table.** Multivariable Mixed-Effects Logistic Regression for Outcome of Magnetic Resonance Imaging Utilization Prior to Biopsy^a^

Characteristic	Overall period (2015-2023)		Year 2023	
	OR (95% CI)	*P* value	OR (95% CI)	*P* value
Age (per 1 y)	1.00 (1.00-1.01)	.03	1.00 (1.00-1.01)	.19
PSA (per 1 ng/dL)	0.99 (0.99-1.00)	<.001	0.99 (0.99-1.00)	<.001
Race				
Asian or Pacific Islander	1.01 (0.88-1.15)	.92	0.90 (0.72-1.23)	.49
Black	0.86 (0.83-0.90)	<.001	0.83 (0.75-0.91)	<.001
White	1 [Reference]	NA	1 [Reference]	NA
Other^b^	0.97 (0.90-1.04)	.40	1.02 (0.87-1.20)	.79
Ethnicity				
Hispanic	1.06 (0.98-1.15)	.15	1.08 (0.91-1.29)	.37
Non-Hispanic	1 [Reference]	NA	1 [Reference]	NA
Rural residence	0.77 (0.74-0.81)	<.001	0.73 (0.67-0.81)	<.001

Abbreviations: NA, not applicable; OR, odds ratio; PSA, prostate-specific antigen.

^a^ Year of biopsy was included as a fixed effect in the overall cohort model but is not shown in the Table.

^b^ Includes the following categories: American Indian or Alaska Native, declined to answer, null, and unknown by patient.

## Discussion

The prebiopsy prostate MRI utilization rate across the VHA increased to 41% through 2023, exceeding the benchmark of 30% in SEER-Medicare, but lower than the 90% utilization rate at some academic health centers.^5^ Despite an equal access system, racial and ethnic and urban-rural disparities in MRI utilization were observed in the VHA, similar to the Medicare population.^3,4^ This may worsen PCa outcomes for Black veterans.^6^

Limitations of this primarily descriptive study include not addressing sources of disparities, nor providing insight into the consequences of MRI utilization. Large datasets are also subject to miscoding. Our findings suggest a need to improve access to prostate MRI nationally and expand capacity within the VHA.

## Notes

Supplement.
Data Sharing Statement
