# Supplementary material for: MRI Utilization Rates for Veterans at Risk of Prostate Cancer
Source: JAMA Netw Open. 2025 Nov 18;8(11):e2543567. doi: 10.1001/jamanetworkopen.2025.43567 (PMC12628099; doi:10.1001/jamanetworkopen.2025.43567)
Supplement: Supplement. — Data Sharing Statement [file jamanetwopen-e2543567-s001.pdf]

Data Sharing Statement

Huang. MRI Utilization Rates for Veterans at Risk of Prostate Cancer. *JAMA Netw Open*. Published November 18, 2025. doi:10.1001/jamanetworkopen.2025.43567

Data
Data available: Yes
Data types: Deidentified participant data, Data dictionary
How to access data: Please email Dr. Hiten Patel and Dr. Mitchell Huang (hitendpatel@gmail.com and mitchmhuang@gmail.com) for inquires about data sharing.
When available: With publication

Supporting Documents
Document types: Statistical/analytic code
How to access documents: See above
When available: With publication

Additional Information
Who can access the data: To researchers interest in the data.
Types of analyses: For research purposes.
Mechanisms of data availability: With investigator support.
